# Fatigue bei chronisch körperlichen Erkrankungen

**DOI:** 10.1007/s00103-024-03951-0

**Published:** 2024-09-16

**Authors:** Joachim Weis

**Affiliations:** Tumorzentrum/Comprehensive Cancer Center Freiburg, Professur Selbsthilfeforschung, Medizinische Fakultät, Universität Freiburg, Hugstetter Str. 49, 79106 Freiburg, Deutschland

**Keywords:** Erschöpfungszustände, Chronisch körperliche Erkrankungen, Depression, Lebensqualität, Diagnostik, Ätiologie, Exhaustion, Chronic health conditions, Depression, Quality of life, Diagnostics, Etiology

## Abstract

Mit dem Begriff Fatigue werden Zustände ungewöhnlicher Müdigkeit und Erschöpfung beschrieben, die in Zusammenhang mit verschiedenen körperlichen Erkrankungen, insbesondere bei Krebs, multipler Sklerose, Parkinson und rheumatoider Arthritis, auftreten können. Im Gegensatz zu Erschöpfungszuständen bei Gesunden ist diese Form der Fatigue dadurch gekennzeichnet, dass sie über längere Zeiträume andauern kann, in keinem angemessenen Verhältnis zu vorangehenden Aktivitäten steht und sich durch Erholungsphasen nicht zurückbildet. Diese Form der Müdigkeit wird als ein multidimensionales Problem beschrieben, das physische, emotionale und kognitive Aspekte umfasst und mit einem hohen subjektiven Leidensdruck verbunden ist. Je nach Ausprägung und Verlauf der Symptomatik führt Fatigue zu einer starken Beeinträchtigung der Lebensqualität und schränkt die Teilhabe und Alltagsbewältigung ein. Ebenso kommt es häufig zu Einschränkungen der Arbeits- und Erwerbsfähigkeit. Wenngleich die Ursachen der Fatigue immer noch nicht vollständig geklärt sind, ist die Fatigue in Zusammenhang mit körperlichen Erkrankungen vor dem Hintergrund eines multifaktoriellen biopsychosozialen Modells zu verstehen. Die Möglichkeiten der Diagnostik und eine Übersicht über verschiedene Verfahren zur Abklärung der Fatigue werden dargestellt. Ebenso werden die Herausforderungen für die medizinische Versorgung skizziert und Hinweise für den Umgang im klinischen Alltag gegeben.

## Abstract (en)

The term fatigue is used to describe states of unusual tiredness and exhaustion that can occur in connection with various chronic diseases, particularly cancer, multiple sclerosis, Parkinson’s disease and rheumatoid arthritis. In contrast to states of exhaustion in healthy people, fatigue is characterized by the fact that it can last for long periods, is disproportionate to previous activities and does not subside after recovery phases. Fatigue is described as a multidimensional problem that encompasses physical, emotional and cognitive aspects and is associated with a high level of subjective suffering. Depending on the severity and course of the symptoms, fatigue is associated with a severely reduced quality of life and it impairs participation and coping with everyday life. It also makes professional reintegration and reemployment more difficult. Although we cannot yet fully explain the aetiology and pathogenesis, fatigue in physical illnesses must be understood based on a multifactorial biopsychosocial model. The possibilities of diagnostics and an overview of various measures for clarifying fatigue are presented. The challenges for medical care are also outlined and advice is given on how to deal with them in everyday clinical practice.

## Einleitung

Müdigkeit und Erschöpfung sind häufige Folgeprobleme, die in Zusammenhang mit verschiedenen chronisch körperlichen Erkrankungen auftreten können und mit dem Fachbegriff Fatigue bezeichnet werden. Vor dem Hintergrund der Komplexität der Fatigue und der zahlreichen biopsychosozialen Einflussmerkmale stellt sie eine große Herausforderung für die medizinische und psychosoziale Versorgung der Patienten dar. Dieser Beitrag gibt einen Überblick über das klinische Erscheinungsbild, das vorliegende Wissen über die Ätiologie und Pathogenese sowie die Möglichkeiten der Diagnostik im Kontext chronisch körperlicher Erkrankungen. Auf die myalgische Enzephalomyelitis bzw. Enzephalopathie/das chronische Fatigue-Syndrom (ME/CFS) wird im Rahmen dieses Artikels nicht eingegangen, da ME/CFS ein eigenständiges Krankheitsbild darstellt. Ebenso wird in diesem Artikel nicht auf die infolge von COVID-19 auftretenden Formen der Müdigkeit (Long-Covid-Syndrom) eingegangen, da ein eigener Beitrag in diesem Heft das Thema fokussiert (siehe Beitrag von Mertens in diesem Themenheft).

## Definition und klinisches Erscheinungsbild

Müdigkeit und Erschöpfung sind alltägliche Erfahrungen, die bei gesunden Menschen in Zusammenhang mit körperlichen oder geistigen Anstrengungen auftreten und in der Regel als vorübergehende Zustände erlebt werden. Nach einer Phase der Erholung oder durch Ausruhen bilden sich diese Zustände wieder zurück und werden daher als normal, teilweise auch als angenehm empfunden. Anders verhält es sich, wenn Menschen mit einer akuten oder chronisch körperlichen Erkrankung Müdigkeit, Kraftlosigkeit oder Erschöpfung erleben. Anders als bei Gesunden erleben Menschen mit einer schweren körperlichen Erkrankung die Müdigkeit als ein stark belastendes Syndrom körperlicher Erschöpfung, Abgeschlagenheit, Schwäche- und Schweregefühl der Muskulatur, das schon bei geringfügigen Anstrengungen auftritt und sich hinsichtlich der Intensität durch Ausruhen oder Schlaf kaum verändern lässt [1]. Bei vielen körperlichen Erkrankungen wird die Fatigue häufig zu einem ständigen Begleiter, der weite Bereiche des Lebens beeinträchtigt und die Lebensqualität sowie die Verrichtungen des Alltags sehr stark einschränkt.

Für diese Zustände der Müdigkeit und Erschöpfung wurde der Fachbegriff Fatigue in die Medizin eingeführt. Müdigkeit ist ein häufiges Symptom [2] bei Erkrankungen wie Krebs [3], multipler Sklerose (MS; [4, 5]), Morbus Parkinson [6, 7], rheumatoider Arthritis (RA; [8, 9]), chronisch obstruktiven Atemwegserkrankungen [10] oder chronischer Niereninsuffizienz [11]. Bei Dialysepatienten mit schweren Symptomen einer Niereninsuffizienz tritt Fatigue eher in fortgeschrittenen Stadien auf [12].

**Abb. 1:**
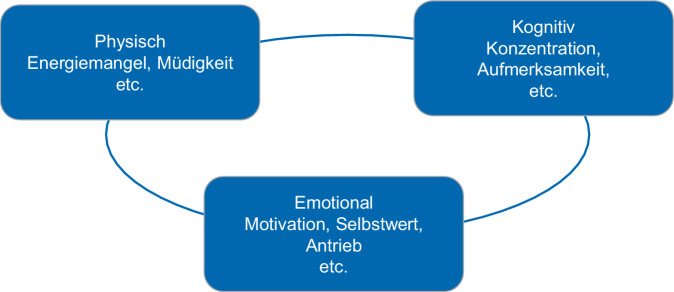
Dimensionen der Fatigue bei körperlichen Erkrankungen [53]

Bei Krebserkrankungen hat sich der Fachbegriff „tumorassoziierte Fatigue“ etabliert, bei MS oder Morbus Parkinson werden in ähnlicher Weise die Begriffe „MS-assoziierte bzw. Parkinson-assoziierte Fatigue“ verwendet. Internationale wissenschaftliche Leitlinien existieren für die tumorassoziierte Fatigue [13–15], für die Fatigue bei Patienten mit RA [9] sowie für die allgemeine Fatigue in Form einer Versorgungsleitlinie der Deutschen Gesellschaft für Allgemeinmedizin [16]. Die meisten Definitionen der Fatigue bei chronisch körperlichen Erkrankungen zeigen eine hohe Übereinstimmung und weisen auf die mehrdimensionale, körperliche, kognitive und emotionale Ausprägung von Fatigue sowie die damit verbundene Beeinträchtigung der Leistungsfähigkeit und der Alltagsfertigkeiten der Betroffenen hin. Auf der körperlichen Ebene kann sich die Fatigue in einer reduzierten körperlichen Leistungsfähigkeit, einem Schwächegefühl, fehlender Ausdauer oder Energiemangel äußern. Auf der emotionalen Ebene sind Antriebs- und Interesselosigkeit, erlebte Frustration sowie fehlende Motivation die häufigsten Symptome, während sich auf der kognitiven Ebene vor allem Probleme in den verschiedenen Formen der Aufmerksamkeit oder im Kurzzeitgedächtnis zeigen können (Abb. 1).

Im Vergleich zur gesunden Normalbevölkerung ohne schwere Erkrankung sind die Fatigue-Werte bei chronisch Kranken deutlich erhöht. So betrug in einer repräsentativen Studie in Großbritannien die Rate ausgeprägter Fatigue bei chronisch Kranken 52 % im Vergleich zu 34 % in der Normalbevölkerung [17, 18]. Bei MS kann die Fatigue als Prodromalsymptomatik bereits vor der Manifestation der Erkrankung auftreten [19, 20] Eine erhöhte Erschöpfbarkeit (Fatigue) ist jedoch auch eines der häufigsten und belastendsten Symptome der MS. Patienten geben eine Abgeschlagenheit und Mattigkeit an, die typischerweise belastungsabhängig oder im Tagesverlauf stärker wird, und beklagen einen Antriebs- und Energiemangel, der die Leistungsfähigkeit in Alltag und Beruf einschränkt. Ebenso zeigt sich bei neurologischen Erkrankungen eine Ermüdung der Muskulatur [21, 22]. Es wird zwischen primären Mechanismen der Fatigue, bspw. neurologischen oder endokrinen Prozessen, und sekundären Mechanismen im Sinne von verstärkenden oder auslösenden Faktoren unterschieden [4].

Laut einer Metaanalyse von 84 Beobachtungsstudien zu Fatigue bei Krebspatienten variieren die Häufigkeitsangaben zwischen 14 % und 100 %, wobei auf der Basis einer gepoolten Datenanalyse die aggregierte Prävalenz auf 52 % geschätzt wird [23]. Die Fatigue bei Krebspatienten kann bei einigen Betroffenen bereits in der Frühphase der Erkrankung, manchmal schon vor der Diagnosestellung auftreten, beginnt aber meist während der Therapie mit wechselnden Intensitäten und kann sich nach dem Ende der Therapie auch wieder zurückbilden. Bei einer weiteren Gruppe treten die Symptome erst nach Abschluss der Therapie auf. Ebenso ist gut belegt, dass die Fatigue auch noch viele Jahre nach erfolgreicher Behandlung einer Tumorerkrankung als eines der Hauptprobleme von Langzeitüberlebenden auftreten kann [24].

Studien, in denen Fatigue zwischen verschiedenen Formen chronischer Erkrankungen untersucht werden, sind vergleichsweise selten. Eine Sekundäranalyse von Querschnittsdaten zur Fatigue von Krebspatienten und Patienten mit RA konnte zeigen, dass RA-Patienten signifikant häufiger unter körperlicher Fatigue und unter stärkeren sozialen Konsequenzen litten [25]. Krebspatienten berichteten dahin gehend signifikant häufiger über kognitive Fatigue. In den emotionalen Aspekten der Fatigue zeigten sich keine Unterschiede.

## Ätiologie und Pathogenese

Es wird davon ausgegangen, dass Fatigue multikausal bedingt ist und sowohl durch die Grunderkrankung als auch durch andere Einflussfaktoren wie Schmerz, gestörter Schlaf oder Folgen körperlicher Inaktivität [26] bedingt ist. Die Ursachen der Fatigue bei chronisch körperlichen Erkrankungen sind auch heute noch weitgehend ungeklärt, wobei sowohl erkrankungsspezifische Faktoren als auch allgemeine physiologische oder psychosoziale Einflussfaktoren diskutiert werden. Wenngleich sich die Fatigue-Symptome in ihren Manifestationen in Abhängigkeit der Grunderkrankung unterscheiden können, wird von übergreifenden Mechanismen der Pathogenese ausgegangen [27–29].

Je nach Art der Erkrankung werden dabei schwerpunktmäßig Veränderungen im Zentralnervensystem (ZNS), der hormonellen Regulation oder des Immunsystems durch die Erkrankung oder Behandlung angenommen. Müdigkeit und Erschöpfung sind Ausdruck für Regulationsvorgänge, die den Organismus vor Überlastungen schützen. Bei neurologischen Erkrankungen werden Läsionen kortikaler und/oder subkortikaler motorischer Bahnen mit Beteiligung des motorischen Kortex bzw. der Basalganglien sowie ein verminderter Energiestoffwechsel im frontalen Kortex diskutiert [21]. Bei der muskulären Erschöpfung lassen sich eine zentrale und eine periphere Ermüdung unterscheiden [30]. Zentrale Ermüdung entsteht durch eine abnehmende Aktivierung der Muskulatur, die durch eine verringerte Aktivität der supraspinalen Motoneurone erklärt werden kann. Eine periphere Ermüdung entsteht durch Prozesse in der neuromuskulären Endplatte, die zu einer Verringerung der Muskelkraft führen kann. Diese physiologisch bedingte Form der Fatigue zeigt sich nicht nur in den Veränderungen der muskulären Leistungsfähigkeit, sondern wird auch durch eine Abnahme kognitiver Prozesse, wie der Aufmerksamkeits- und Konzentrationsfähigkeit, begleitet.

Nach aktuellen, jedoch immer noch kontrovers diskutierten Erkenntnissen geht man davon aus, dass die 3 Systeme des menschlichen Organismus, das ZNS, das endokrine System sowie das Immunsystem, miteinander interagieren und dadurch komplexe Regulationsprozesse in Gang gesetzt werden [31, 32] und mit Prozessen innerhalb der zentralen Aktivierungssysteme zusammenhängen, die für die Handlungssteuerung verantwortlich sind [33, 34].

Hierbei werden verschiedene somatische und psychologische Erklärungsmodelle diskutiert, die zwar noch nicht umfassend empirisch geklärt sind, aber für das Verständnis und als Anregung für die Grundlagen- und Therapieforschung hilfreich sind. 2 zentrale somatopsychische Erklärungsmodelle fokussieren die kognitiv-behavioralen Prozesse und die Regulation von Stress [35] und sollen hier kurz vorgestellt werden. Kognitiv-behaviorale Modelle gehen davon aus, dass psychogene Faktoren, wie Wahrnehmungsverzerrungen, dysfunktionale Überzeugungen oder Einstellungen und inadäquate Problemlösungen, die wesentlichen Ursachen für die Entstehung und Aufrechterhaltung persistierender Fatigue sind und biologische bzw. somatische Funktionsstörungen eine geringere Rolle spielen. Der Einfluss von kognitiv-behavioralen Faktoren wird primär für die Entstehung von ME/CFS, aber auch für die Fatigue bei chronischen Erkrankungen diskutiert [36]. Hierbei wird angenommen, dass Anpassungsprozesse und Reaktionen der Betroffenen auf die erlebte Fatigue dazu führen, dass die Symptome persistieren und zu einem andauernden Syndrom werden. Das Erleben der Fatigue führt zu physiologischen und psychosozialen Veränderungen, die als aufrechterhaltende Faktoren der Fatigue zu einer fortschreitenden Abwärtsspirale des Krankheitsgeschehens beitragen können. Allerdings müssen diese Vorstellungen um den Einfluss biologisch-somatischer Faktoren bei körperlichen somatischen Erkrankungen erweitert werden [37].

Da körperliche Erkrankungen für die Betroffenen mit vielfältigen Belastungen verbunden sind, könnte die komplexe Entstehung von Fatigue auch in einem Modell der psychobiologischen Stressregulation (Stress-Allostase-Modell) erklärt werden [38]. Ausgangspunkt für dieses Modell ist die Annahme, dass Menschen verschiedenen Stressfaktoren auf somatischer, psychischer und sozialer Ebene ausgesetzt sind, die eine Anpassungsreaktion erfordern und im Falle von chronischer Überforderung zur Entstehung von Krankheiten führen können [39]. Als Allostase werden in diesem Modell physiologische Prozesse der Anpassung an die stressbedingten Belastungs- und Anforderungssituationen verstanden, um seine Funktionalität zu erhalten. Die Prozesse der Allostase zielen darauf ab, die Stabilität des Organismus durch fortwährende Adaptationsvorgänge in den verschiedenen Systemen zu gewährleisten. Bei der Vermittlung der physiologischen Reaktionen spielen verschiedene Biomarker eine Rolle. Dies sind insbesondere die Hormone der Hypothalamus-Hypophysen-Nebennieren-Achse (HHN-A) und des eng damit verbundenen autonomen Nervensystems als primäre Stressmediatoren sowie weitere Mediatoren, wie Zytokine und Neurotransmitter [40]. Eine Metaanalyse von 267 Studien zum Zusammenhang biologischer Marker der Stressregulation und Entstehung von Krankheiten konnte zeigen, dass chronische Überlastungen mit einem schlechteren Gesundheitszustand assoziiert sind [41]. Demzufolge lassen sich in einem Stress-Allostase-Modell Fatigue-Symptome als das pathophysiologische Korrelat einer Erschöpfung der adaptiven Ressourcen des Körpers durch andauernde somatische, psychische oder soziale Stressoren infolge chronisch körperlicher Erkrankung verstehen.

## Die Bedeutung psychosozialer Einflussfaktoren

Wie bereits erläutert, wird Fatigue bei chronisch körperlichen Erkrankungen vor dem Hintergrund eines biopsychosozialen Modells verstanden. Hierbei lassen sich vielfältige Zusammenhänge sowie Wechselwirkungen mit verschiedenen Merkmalen des psychischen Befindens feststellen. In zahlreichen Studien werden konsistent Assoziationen mit psychosozialen Faktoren und seelischen Störungen aufgezeigt, wobei Depressionen sowie Angststörungen im Vordergrund stehen [42–44].

Überschneidungen zwischen Depression und Fatigue zeigen sich in den körperlich-vegetativen Leitsymptomen wie Antriebslosigkeit, Abgeschlagenheit, Energielosigkeit sowie vermindertes Interesse an alltäglichen Aktivitäten, während das Vorhandensein psychischer Symptome, wie bspw. negatives Selbstwertgefühl oder Schuldgefühle, einen deutlichen Unterschied darstellen. Auf der kognitiven Ebene sind Einschränkungen der Konzentrationsfähigkeit bei beiden Störungsbildern gegeben. Fatigue kann zusammen mit einer leichten oder mild ausgeprägten Depression sowie subsyndromalen depressiven Verstimmungen einhergehen, jedoch auch ohne derartige Symptome auftreten. Ebenso kann eine unbehandelte oder nicht erfolgreich behandelbare Fatigue im Verlauf eine depressive Störung nach sich ziehen. In Studien mit Parkinson-Patienten konnten klare Zusammenhänge zwischen Fatigue und Depression aufgezeigt werden [45, 46]. In einer Querschnittstudie konnte gezeigt werden, dass die Schwere der Erkrankung die Symptome der Depression direkt beeinflusste, während die depressive Symptomatik einen direkten Einfluss nur auf Teilaspekte der Fatigue (reduzierte Motivation) hatte [46]. Bei Studien zur tumorassoziierten Fatigue fand sich, dass neben der Depression auch Angst sowie Strategien der Krankheitsverarbeitung wie Vermeidung, Ablenkung oder problemlösungsorientierte Strategien das Erleben der Fatigue beeinflussen können [47]. In einer Querschnittstudie mit MS-Patienten waren höhere Depressionswerte sowie Angstwerte mit Fatigue assoziiert [48]. Bei Patienten mit Morbus Parkinson waren Verarbeitungsstrategien in Richtung der Bewegungsvermeidung entscheidende Trigger für die Ausprägung der Fatigue [49].

Aus diesen Befunden lässt sich schlussfolgern, dass die Fatigue bei chronisch körperlichen Erkrankungen insbesondere in Bezug auf Depression, Schlafstörungen sowie Schmerzsymptomatik differenzialdiagnostisch abgeklärt werden sollte [49–52].

## Diagnostisches Vorgehen

Die Fatigue im Kontext von körperlichen Erkrankungen ist im Klassifikationssystem der Erkrankungen (ICD [International Statistical Classification of Diseases and Related Health Problems]) bisher nicht als eigenständige Krankheitskategorie enthalten, obwohl sie durch die mit ihr verbundenen somatischen und psychischen Auswirkungen sowie sozialen Belastungen einen ausgeprägten Krankheitswert darstellen kann. Es gibt jedoch Möglichkeiten, die Symptomatik in der ICD zu kodieren. So wird in der englischen Ausgabe der ICD-10-CM die tumorassoziierte Fatigue seit 2016 als „R 53.0 Neoplastic (malignant) related fatigue“ kodiert. In der deutschen Version (ICD-10-GM-2022) findet sich ein Code (R 53) für „Unwohlsein und Ermüdung“ unter „Allgemeinsymptome“. In der Entwurfsversion der deutschen ICD-11 gibt es „Fatigue“ mit dem Code MG22 unter „Allgemeinsymptome oder klinische Befunde“.

**Abb. 2:**
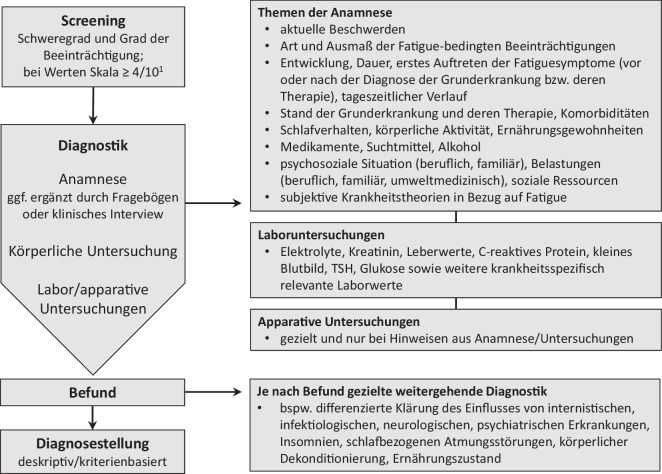
Algorithmus zur Diagnostik der Fatigue. ^1^Numerische Analogskala: 0 = keine Fatigue, 1–3 = milde Fatigue, 4–6 = moderate Fatigue, 7–10 = schwere Fatigue. Bei Werten ≥ 4 wird eine weiterführende Diagnostik durchgeführt. *TSH* Thyroidea stimulierendes Hormon. (Leicht modifiziert nach [53])

Die Hauptziele der Diagnostik der Fatigue bei einer zugrunde liegenden körperlichen Erkrankung sind, das Beschwerdebild und die möglichen primären und sekundären Einflussfaktoren zu erfassen. Hierbei ist zwischen den direkt in Verbindung mit der Erkrankung stehenden Beschwerden und sekundären Folgeproblemen zu unterscheiden. Neben der genauen Erfassung der Fatigue-bezogenen Beschwerden schließt sie auch die Einschätzung der möglichen Beeinträchtigungen der Lebensqualität, der Funktionsfähigkeit und der Teilhabe ein. Die vorliegenden Leitlinien [9, 13–16] empfehlen zur Diagnostik der Fatigue ein gestuftes, interdisziplinär abgestimmtes, multimodales Vorgehen, das sich an dem Schweregrad der Symptomatik und deren Auswirkungen orientiert. An erster Stelle des diagnostischen Prozesses steht ein Screening zur Selbsteinschätzung der Fatigue mit einer einfachen Skala. Das vom National Comprehensive Cancer Network (NCCN) für den Bereich der tumorassoziierten Fatigue entwickelte Screening-Instrument kann auch bei Patienten mit anderen körperlichen Erkrankungen eingesetzt werden [13]. Dieses Screening erlaubt über eine Abstufung von 0 = keine bis 10 = stärkste vorstellbare Fatigue eine Einteilung in milde, moderate und stark ausgeprägte Fatigue. Ist die Fatigue moderat oder stark ausgeprägt (Werte ≥ 4), wird eine vertiefende Diagnostik empfohlen. Abb. 2 zeigt einen Algorithmus für eine abgestufte Diagnostik, die für die tumorassoziierte Fatigue vorgestellt wurde [53] und modifiziert auch für andere chronisch körperliche Erkrankungen angepasst werden kann.

An zentraler Stelle steht die Anamnese über die Selbsteinschätzung und Beschreibung der körperlichen, psychischen und sozialen Dimensionen der Symptomatik durch die Betroffenen, ergänzt durch eine körperliche Untersuchung und gezielte Labor- und apparative Diagnostik. Hierbei ist zu beachten, dass Fatigue eine subjektive Wahrnehmung darstellt, die nur bedingt von außen beobachtet oder gemessen werden kann. Ebenso müssen im Prozess der Diagnosestellung einer Fatigue sorgfältig potenzielle somatische und psychiatrische sowie pharmakogene Ursachen abgeklärt werden.

Neben der Abklärung vorangegangener Infektionserkrankungen oder bestehender chronischer Erkrankungen werden in den genannten Leitlinien folgende Hinweise zur anamnestischen Erhebung genannt:

- Erfassung der zeitlichen Entwicklung, Qualität, Dauer bzw. des (tages‑)zeitlichen Verlaufs sowie des Schweregrads der Fatigue-Symptomatik,
- Differenzierung der allgemeinen Erschöpfung im Hinblick auf Schläfrigkeit und andere Formen von Schlafstörungen,
- Abklärung möglicher neuromuskulärer Erkrankungen oder Störungen motorischer Abläufe bzw. Funktionen,
- Abklärung psychosozialer Einflussfaktoren und Differenzialdiagnostik psychischer Komorbidität,
- funktionelle Beeinträchtigung (Mobilität, Familie, Beruf) und damit assoziierte Veränderungen der Lebenssituation,
- Erfassung der subjektiven Vorstellungen des Patienten zur Ätiologie des Symptoms und damit verbundene Befürchtungen,
- Erfassung der familiären, beruflichen und sozialen Situation (aktuell und biografisch),
- somatische/vegetative Anamnese im Hinblick auf die Funktion von Organsystemen im Sinne einer vegetativen Anamnese (kardial, pulmonal, gastrointestinal, urogenital, ZNS, Haut, Gelenke), aktueller Body-Mass-Index (BMI)/Gewichtskategorie, Gewichtsveränderungen,
- Bestimmung der von Ärzten verordneten Medikation bzw. Selbstmedikation, sonstige psychotrope Substanzen,
- arbeits- und umweltmedizinische Basisanamnese bestehender oder früherer beruflicher oder umweltbedingter Belastungen.

Zur differenzierten Erfassung der Fatigue liegt eine Reihe von standardisierten ein- oder mehrdimensionalen Fragebögen vor. Man unterscheidet generische Fragebögen, die diagnoseübergreifend eingesetzt werden können, und diagnosespezifische Verfahren, wobei viele generische Verfahren auch diagnosespezifisch eingesetzt werden. Häufig eingesetzte generische Instrumente sind die Fatigue-Severity-Scale (FSS; [54]) oder die Fatigue-Skala (FS; [55]), die auch als deutschsprachig standardisierte Verfahren vorliegen [56]. Eine Zusammenstellung verschiedener generischer Verfahren findet sich bei Hjollund et al. [57]. Eine Übersicht über deutschsprachige standardisierte Instrumente zur Erfassung der tumorassoziierten Fatigue haben Weis et al. [53] zusammengestellt. Fatigue kann auch über die entsprechenden Subskalen generischer Instrumente zur Erfassung der Lebensqualität erhoben werden, allerdings zumeist nur in ihrer physischen Ausprägung, wie bspw. über die SF12/SF36 Subskala Vitalität/Erschöpfung. Darüber hinaus gibt es eine Reihe von Verfahren zur spezifischen Erfassung der Fatigue bei neurologischen Erkrankungen [5, 58].

Aufgrund der im vorangehenden Abschnitt beschriebenen Überschneidung zwischen Fatigue und Depression ist eine differenzialdiagnostische Abklärung bzw. Abgrenzung erforderlich [43]. Für den klinischen Alltag bietet der sogenannte „Zwei-Fragen-Test“ (PHQ-2) eine einfache Möglichkeit, das Vorliegen einer depressiven Störung mit einer hohen Sensitivität (96 %) festzustellen. Fragebogeninstrumente wie die Hospital Anxiety und Depression Scale (HADS), Personal Health Questionnaire, Kurzversion Depression (PHQ 9) oder General Anxiety Disorder Scale (GAD7) können als Screening-Verfahren eingesetzt werden, erlauben aber keine eindeutige Diagnose einer psychischen Störung. Alle genannten Fragebögen liegen in deutschsprachig validierten Versionen vor [59]. Im Falle eines positiven Screenings mithilfe der genannten Fragebögen ist eine diagnostische Sicherung über ein standardisiertes klinisches Interview (CIDI in Anlehnung an ICD oder SKID in Anlehnung an DSM) erforderlich.

## Fazit

Das Fehlen von objektiven Tests, mit denen die Fatigue gemessen werden kann, trägt wesentlich dazu bei, dass das Beschwerdebild vom Umfeld der Betroffenen, auch dem der professionell Behandelnden, nicht immer verstanden wird und mit einem nicht unerheblichen sozialen Stigma verbunden sein kann. Viele Untersuchungen zeigen, dass Fatigue selten erfragt wird. Dies geschieht häufig deshalb, weil sie als normale und wenig beeinflussbare Begleiterscheinung körperlicher Erkrankungen angesehen wird. Trotz der zunehmenden Aufmerksamkeit für das Thema Fatigue in den letzten beiden Jahrzehnten, insbesondere bei Krebs und neurologischen Erkrankungen, nehmen die Behandelnden das Ausmaß der Belastungen und Einschränkungen der Betroffenen durch Fatigue noch immer nicht ausreichend wahr und unterschätzen die Behandlungsbedürftigkeit [21, 24, 60]. Gründe für eine unzureichende Kommunikation über Fatigue finden sich sowohl aufseiten der Betroffenen als auch bei den Behandelnden. Viele Patienten sprechen die Beschwerden nicht an, da sie nicht als klagsam erscheinen möchten, sie diese als zur Krankheit oder Therapie gehörend betrachten oder einfach nicht aktiv danach gefragt werden [61, 62]. Auch befürchten viele, dass die Symptome einen Rückfall oder die Progredienz der zugrunde liegenden Erkrankung bedeuten könnten [63, 64]. Aufseiten des Behandelnden sind mangelnde Zeit und fehlende Kenntnisse zur Diagnostik und zu Behandlungsmöglichkeiten wichtige Hinderungsgründe für eine angemessene Kommunikation [60]. Nicht zuletzt ist es aufseiten der Ärzte ein weiterer Grund, dass sie die krankhaft erlebte Fatigue bei chronisch körperlich Kranken nicht hinreichend von der Alltagsmüdigkeit abgrenzen können und die Beschwerden der Betroffenen dadurch nicht ernst nehmen.

Häufig blicken Ärzte bei körperlichen Erkrankungen auf körperliche Ursachen, während psychosoziale Einflussfaktoren oft zu wenig in Betracht gezogen werden. Gerade bei körperlichen Erkrankungen sind die somatischen Ursachen der Fatigue oft nicht eindeutig bestimmbar. Deshalb sollte bei der Diagnostik der Fatigue von Beginn an ein biopsychosoziales Verständnis mit den Betroffenen erarbeitet und der aktuelle Kenntnistand vermittelt werden. Bei Vorliegen einer chronisch körperlichen Erkrankung wird die Müdigkeit oft vorschnell auf den Krankheitsprozess selbst bezogen. Wie dargestellt, sind psychische Verarbeitungsstrategien als Reaktionen auf die Fatigue, Depression, Schlafstörungen, Schmerzen, Folgen körperlicher Inaktivität, Neben- oder Folgewirkungen der Therapie in ihren Wechselwirkungen bedeutsam und deren Bestimmung notwendig. Für Betroffene sowie Ärzte kann es dabei auch vorkommen, dass vorschnell unzureichend belegte (Pseudo‑)Diagnosen auf somatischer Ebene, bspw. in Richtung Eisen- oder Vitamin-D-Mangel, Hypotonie oder Hypoglykämie, vorgebracht werden. Aufgrund der Angst vor einer psychischen Stigmatisierung fühlen sich gerade Patienten mit körperlichen Erkrankungen häufig mit solchen Diagnosen ernst genommen und entlastet [63]. Dieser Artikel soll dazu beitragen, die Fatigue bei vorliegenden körperlichen Erkrankungen in ihrer Komplexität besser verstehen zu können und die Klagen der Patienten ernst zu nehmen sowie diese nicht vorschnell somatisch oder psychisch im Sinne einer unzureichenden Verarbeitung oder nicht gelingenden psychischen Anpassung zu etikettieren. Vorhandene Leitlinien geben hierzu für die verschiedenen Krankheitsbilder eine wichtige Orientierung im Hinblick auf die diagnostischen und therapeutischen Möglichkeiten.
